# Epidermal growth factor receptor T790M mutation as a prognostic factor in EGFR-mutant non-small cell lung cancer patients that acquired resistance to EGFR tyrosine kinase inhibitors

**DOI:** 10.18632/oncotarget.19681

**Published:** 2017-07-29

**Authors:** Guangzhi Ma, Jing Zhang, Hai Jiang, Nannan Zhang, Liyuan Yin, Wen Li, Qinghua Zhou

**Affiliations:** ^1^ Lung Cancer Center, West China Hospital, Sichuan University, Chengdu 610041, P. R. China; ^2^ Department of Thoracic Surgery, West China Hospital, Sichuan University, Chengdu 610041, P. R. China; ^3^ Department of Neurosurgery, West China Hospital, Sichuan University, Chengdu 610041, P. R. China; ^4^ Department of Orthopedic Surgery, West China Hospital, Sichuan University, Chengdu 610041, P. R. China

**Keywords:** NSCLC, T790M, EGFR-TKIs, prognosis, meta-analysis

## Abstract

Epidermal growth factor receptor (EGFR) T790M mutation accounted for over half of drug resistance cases in EGFR-mutant non-small cell lung cancer (NSCLC) patients treated with EGFR tyrosine kinase inhibitors (TKIs) and led to different outcomes. This study aimed to assess the prognostic role of T790M in NSCLC patients treated with EGFR-TKIs that developed drug resistance. Eligible literatures were reviewed from various databases and a meta-analysis was performed to evaluate the prognostic role of T790M mutation in EGFR-TKIs treated patients that went progression. Three studies containing 192 patients were included in the meta-analysis. The pooled hazard ratios (HRs) for overall survival (OS) and progression-free survival (PFS) were 0.66 (95% CI 0.49–0.89, *P* = 0.007) and 0.53 (95% CI 0.35–0.79, *P* = 0.002) respectively. Subgroups analyses were also performed on OS and PFS according to patients’ districts, gender and histological type. In conclusion, T790M as a common mutation to cause drug-resistance in EGFR-TKIs treated NSCLC patients may be a favorable prognostic factor on OS and PFS both. Further studies are necessary to demonstrate the prognostic role of secondary T790M in NSCLC patients.

## INTRODUCTION

Lung cancer is and continues to be the leading cause of cancer death globally, accounting for over 1.1 million cancer deaths annually [1, 2]. It is also the leading cause of cancer death in men and the second leading cause of cancer death in women (after breast cancer), with approximately 1.8 million new cases reported annually worldwide [3]. Statistically 85% of lung cancer cases were non-small cell lung cancer (NSCLC) according to pathology type [4]. Besides its high incidence, the prognosis of NSCLC remains poor, with a 5-year survival rate around 15% in Europe and USA [1, 5]. To patients with advanced NSCLC, treatment strategies include adjuvant radiotherapy, combined chemotherapy and first-line target-therapy [6].

Target-therapy with epidermal growth factor receptor tyrosine kinase inhibitors (EGFR-TKIs), were proven an effective choice for NSCLC patients with EGFR mutations (L858R or deletion in exon 19) [7, 8]. Some studies indicated that patients with advanced NSCLC who underwent EGFR-TKIs had more favorable outcomes as first-line treatment compared to chemotherapy [9–11]. The response rate was over 70% in all EGFR-mutated NSCLC cases [8]. Clinical data showed the overall survival (OS) of EGFR-TKIs treated NSCLCs was 2 to 24 months while the progression-free survival (PFS) was between 6 to 12 months [8, 12]. However, drug resistance eventually occurred to almost every EGFR-TKIs treated NSCLC patient within 10 months period after initial drug use, and hardly evitable [13, 14]. Although the mechanism of EGFR-TKIs resistance remains intricate in many cases, the threonine-to-methionine substitution in EGFR gene at codon 790 (T790M) resulted in 50% of NSCLC patients who developed TKIs drug resistance [15]. T790M in exon 20 of EGFR gene was first reported by Kobayashi et al, and then proved to cause drug resistance in NSCLC patients treated with EGFR-TKIs [16]. How such mutation emerged in EGFR-TKIs treated NSCLC patients remain controversial. Although de novo T790M was detectable in some pretreatment cases, but was rare while high sensitive procedures were required to perform assays [17, 18]. On the other hand, the vast majority of advanced NSCLC patients were found to harbor T790M mutation after a period of TKIs use, therefore most researchers tended to believe T790M mutation was more an acquired mutation [19]. Since T790M mutation could cause drug resistance in NSCLC patients treated with EGFR TKIs, the existence of such mutation should associate with patients’ prognosis. Oxnard et al. first reported that patients with acquired T790M mutation had relatively favorable outcomes compared to those without in NSCLC patient that developed EGFR-TKIs resistance [20]. Ji et al. found that NSCLC patients after progression on TKIs with acquired T790M mutation had better outcome on PFS, however no significant relation was found between OS and prognosis [21]. Interestingly, according to a study conducted by Zheng et al, T790M led to poorer outcomes on OS in advanced NSCLC EGFR-TKIs treated patients that underwent progression [22].

Due to those inconsistent conclusions above, we herein aimed to perform a meta-analysis to explore the prognostic role of T790M mutation in advanced NSCLC patients treated with EGFR-TKIs that developed drug resistance.

## RESULTS

### Study selection

A total of 566 studies were drawn from the initially search for eligible studies. Titles and abstracts were screened by the reviewers of each identified literature. Studies were excluded for the following reasons: duplicate studies (*n =* 17), studies on animals (*n =* 15), laboratory studies such as signal pathways or molecular mechanisms (*n =* 267), reviews (*n =* 125) and case reports (*n =* 114). Full text of the 28 potential studies were retrieved and reviewed. 19 of the remained studies were then further excluded: 8 studies focused on the correlation between pretreatment T790M and prognosis of EGFR-mutant advanced NSCLC patients, 5 of the studies were based on assay methods, 4 studies evaluated different index such as response rate and 5-year survival, 2 studies had insufficient/invalid data, and 3 were excluded for evaluating T790M status through plasma DNA. 6 eligible literatures [20, 21, 23–26] were further reviewed, and were 3 of which were removed: 1 study had early stage patients such as stage I to receive TKIs [26], 1 study had patients whose T790M status were inconsistent between mutation assays [25], and 1 study observed survival of patients that received TKIs after progression from TKI [23]. In all, 3 literatures eventually matched our criteria of inclusion for final meta-analyses. Two studies scored 7 [21, 24] and one scored 8 [20] according to Newcastle–Ottawa Scale (NOS) criteria [27] in methodological assessment.

**Figure 1:**
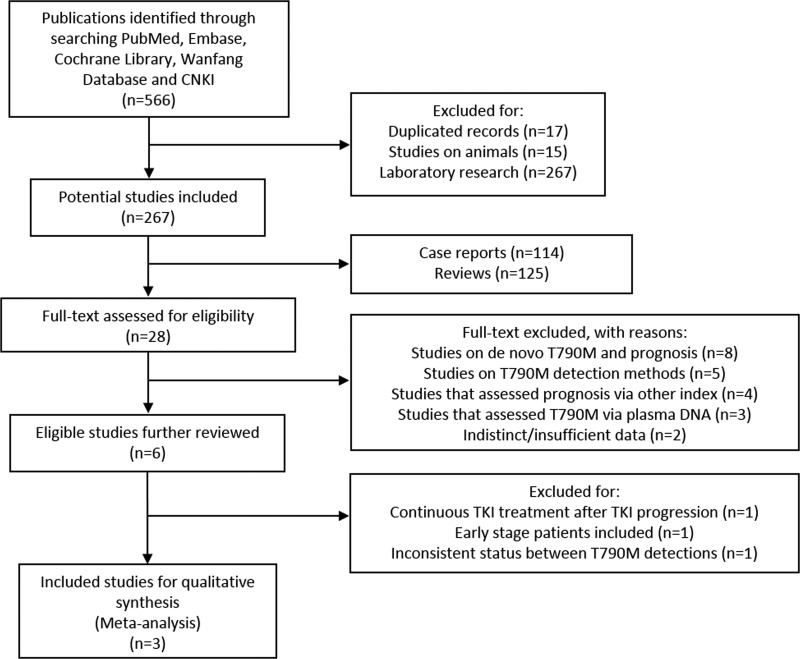
Selection process for eligible studies

The process of publication selection was shown in Figure 1.

### Study characteristics

Among the 3 eligible studies, 2 were from Asia, and the 1 were from USA. Altogether 192 patients (133 female and 59 male) were included in the study. All participants were diagnosed NSCLC with EGFR-mutation and eventually acquired drug resistance after a period of EGFR-TKIs therapy. EGFR T790M mutation was found in 107 patients from tumor tissue biopsy. All included patients had advanced lung cancer and among which adenocarcinoma were the most common histological type. Smoking history status was found in 2 studies with 1 study missing. The EGFR TKIs involved were mostly gefitinib and erlotinib, however one study study had 3 cases that underwent afatinib. To conclude, the basic information of eligible studies was provided in Table 1.

**Table 1:** The characteristics of the included publications

First author	Year	Country	N (F/M)	N with T790M mutation(F/M)	Smoking history (never/smoker)	Clinical stage	Specimen	Histology	EGFR-TKIs received	Quality score
Oxnard	2011	USA	93(60/33)	58	61/32	IV(71)/Recurrent(22)	Biopsy	NSCLC	Erl.(64)/Gef.(29)	8
Ji	2013	Korea	26(16/10)	11(7/4)	—	—	Biopsy	Adcc(25)/Sqcc(1)	Gef.	7
Matsuo	2016	Japan	73(57/16)	38	56/17	Advanced(53)/Recurrent(20)	Biopsy	Adcc(72)/Sqcc(1)	Erl.(12)/Gef.(58)/Afa.(3)	7

N: Number of patients; F: Female; M: Male; EGFR-TKIs: Epidermal growth factor receptor tyrosine kinase inhibitors; NSCLC: Non-small cell lung cancer; Adcc: Adenocarcinoma; Sqcc: Squamous cell carcinoma; Erl: Erlotinib; Gef: Gefitinib; Afa: Afatinib.

### Meta-analysis results

**Figure 2:**
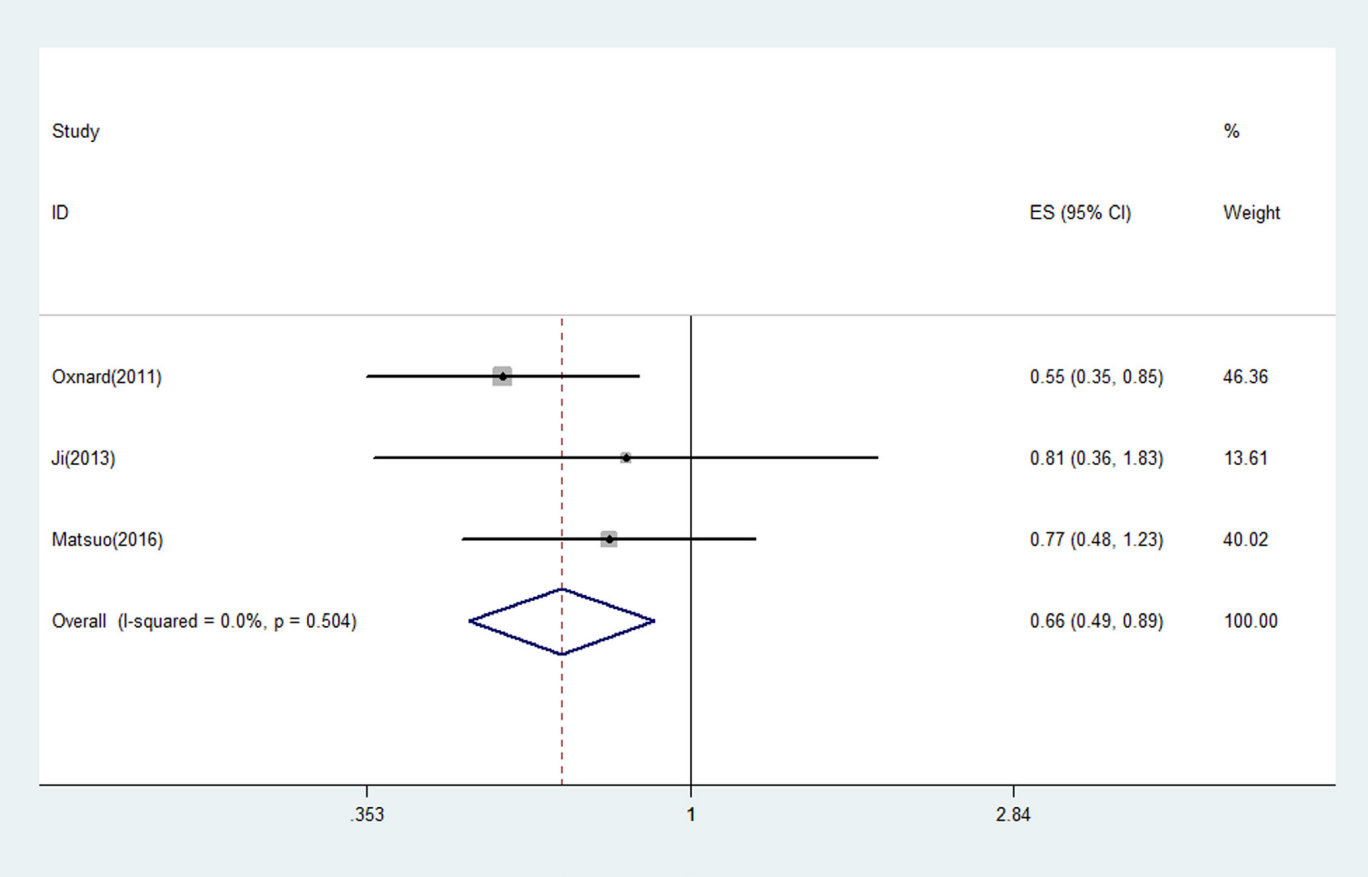
The pooled estimated survival (ES) (hazard ratio) for OS in EGFR-TKIs treated NSCLC patients with acquired T790M that went progression

The prognostic role of acquired T790M mutation was assessed by survival time including OS and PFS. PFS was examined in 2 studies [21, 24], and the pooled HR was 0.53 (95% CI 0.35–0.79, *P* = 0.002), indicating T790M mutation was associated with better outcome on PFS (Figure 2). The heterogeneity was not significant (I^2^ = 33.3%, *P =* 0.221) and fixed-effects model was used for calculation.

**Figure 3:**
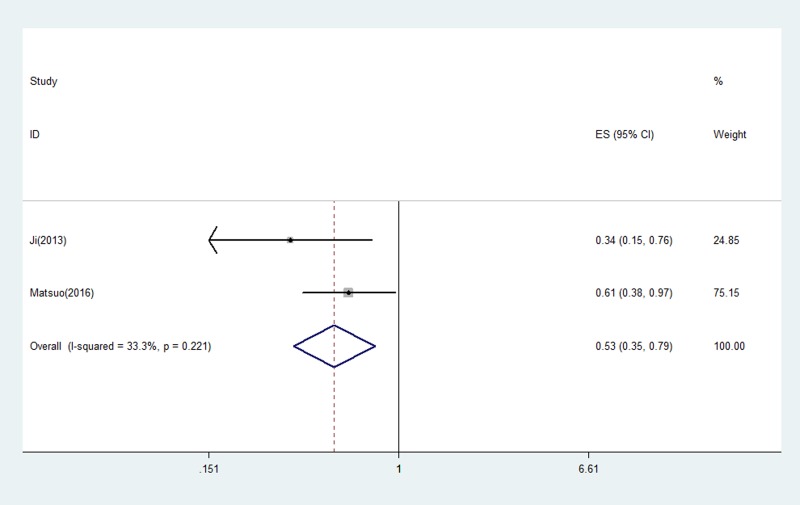
The pooled estimated survival (ES) (hazard ratio) for PFS in EGFR-TKIs treated NSCLC patients with acquired T790M that went progression

All 3 eligible studies discussed the correlation between acquired T790M and OS. The pooled HR for OS was 0.66 (95% CI 0.49–0.89, *P* = 0.007) (Figure 3). The heterogeneity was not statistically significant (I^2^ = 0.0%, *P* = 0.504) therefore fixed-effects model was used to pool data.

### Subgroup analysis

Subgroups were divided due to varied districts (Asian), gender, and histology. Among subgroup results were statically significant (*P* < 0.05).

### Asian

Two studies were Asian studies that were from Japan and Korea. Both studies provided PFS and OS. Therefore combined HR for PFS in Asian was same as PFS value mentioned above. Combined HR and 95% CI for OS in Asian were 0.78 (0.52–1.17).

### Gender

When female patients were the majority of all patients involved (> 50%), the pooled HRs for PFS and OS were 0.53 (95% CI 0.35–0.79, *P* = 0.02, I^2^ = 33.3%) and 0.66 (95% CI 0.49–0.89, *P* = 0.007, I^2^ = 0.0%).

### Histology

Adenocarcinoma was the most common pathological type of all included studies. When adenocarcinoma was over 75%, the pooled HR for PFS and OS were 0.53 (95% CI 0.35–0.79, *P* = 0.002, I^2^ = 33.3%) and 0.78 (95% CI 0.52–0.89, *p* = 0.909, I^2^ = 0.0%). The heterogeneity in neither of the subgroups was significant.

All pooled results were displayed on Table 2.

**Table 2:** Meta-analyses of EGFR T790M and survival outcomes of EGFR-mutant NSCLC patients treated with EGFR TKIs that acquired drug resistance

	N of studies	Model	HR (95% CI)	Log-rank *P*	Heterogeneity (p, I^2^)	Conclusion
Total PFS	2	Fixed	0.53(0.35–0.79)	0.002	0.221, 33.3%	Positive
Asian PFS	2	Fixed	0.53(0.35–0.79)	0.002	0.221, 33.3%	Positive
Female > 50% PFS	2	Fixed	0.53(0.35–0.79)	0.002	0.221, 33.3%	Positive
Adcc > 75% PFS	2	Fixed	0.53(0.35–0.79)	0.002	0.221, 33.3%	Positive
Total OS	3	Fixed	0.66(0.49–0.89)	0.007	0.504, 0.0%	Positive
Asian OS	2	Fixed	0.78(0.52–1.17)	0.234	0.909, 0.0%	Negative
Female > 50% OS	3	Fixed	0.66(0.49–0.89)	0.007	0.504, 0.0%	Positive
Adcc > 75% OS	2	Fixed	0.78(0.52–0.89)	0.234	0.909, 0.0%	Negative

PFS: progression-free survival; OS: overall survival; Adcc: adenocarcinoma; N: number; HR: hazard ratio; CI: confidence interval.

### Publication bias

**Figure 4:**
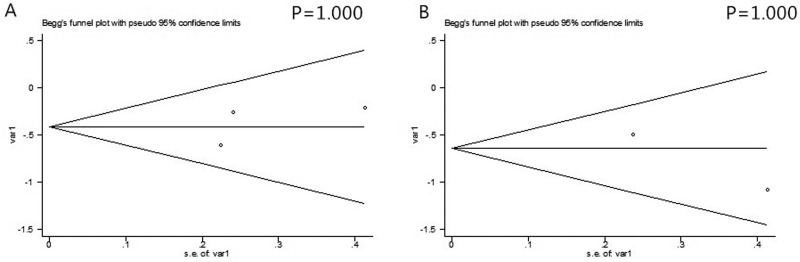
The Begg’s publication bias plots of the studies that reported the correlation between secondary T790M mutation and OS (**A**) and PFS (**B**) in EGFR-TKIs treated NSCLC patients that acquired drug resistance.

As shown in the plots of publication bias in Figure 4, publication bias was not found in this meta-analysis.

## DISCUSSION

The aim of the present study was to discuss the prognostic role of secondary T790M mutation in NSCLC EGFR-TKIs resistant patients. A meta-analysis was performed and the data were pooled. As a result, among EGFR-TKIs treated patients that acquired drug resistance, those who bore T790M had a better outcome on OS and PFS both compared with T790M naïve patients. According to subgroup analyses, the prognostic roles of acquired T790M were also indicative and favorable prognosis was found in Asian patients when T790M co-existed. Pathology analysis suggested that patients with adenocarcinoma might led to better outcomes when T790M co-existed as well. When female patients were more than 50%, T790M seemed to be a favorable predictor on PFS, whereas no significant correlation was found between acquired T790M and OS. Thus gender constituent ratio seemed to have an impact on survival of patients with acquired T790M mutation. However due to lack of original data we weren’t able to compare the direct relationship between genders.

Seemingly EGFR T790M mutation in NSCLC patients that caused EGFR-TKIs drug resistance could led to poorer prognosis, yet the pooled results suggested the opposite. Zheng et al. [22] reported an adverse impact on survival (OS) when T790M was positive, their study assessed T790M status by checking plasma DNA. However the lack of real-time matched tumor tissues to compare with plasma T790M was a major deficiency and the sensitivity to detect plasma T790M has been questioned [28]. Hence considering the heterogeneity that plasma DNA might cause, all studies without NSCLC tissue biopsy were excluded in the current meta-analysis. Another three studies [23, 25, 26] that reported the survival of NSCLC patients that acquired T790M by identifying tumor tissue were also removed for meta-analysis. Uramoto et al. [26] reported a favorable survival of Japanese patients with T790M presence. However some of their included early participants such as stage IA/IB and TKIs were not appropriate to be applied to these patients, therefore the literature was excluded for the current study. Li et al. [23] focused on the survival of patients after TKIs progression. Although a better outcome were also found among TKIs-resistant patients that harbored T790M, all patients that were involved in their study received continuous EGFR-TKIs even after progression, hence this study was also excluded. Likewise, Kuiper et al. [25] found patients with acquired T790M had longer PFS and OS treated with EGFR-TKIs. Yet the detection results among some of their participants were inconsistent, some T790M-positive cases at first post-TKI biopsy eventually became T790M-negative in later re-biopsies. Therefore, their studies were also ineligible to be pooled in this meta-analysis.

To account for the results that patient with and without T790M mutation after progression on TKI had inconsistent outcome, several factors should be considered. Indolent characteristics of tumor cells that harbored T790M mutation are the most probable mechanism that might explain, yet correspondent laboratory evidence remains insufficient [20, 29]. Tumor heterogeneity also played an important role in the progression of oncogene-driven EGFR-TKIs treated cancers [25]. Other mechanisms (c-met etc.) besides T790M mutation that caused resistance in NSCLC could associate with earlier metastasize and worse tumor behavior, which eventually resulted in shorter survival [20, 21]. In addition, most patients were given combined subsequent chemo-therapy to treat the resistant clones of tumor cells, and these cells was more sensitive to cytotoxic drugs compared with TKIs-resistant cells without T-790M mutation [20, 30].

It is important to have a better understanding of the emergence T790M mutation in NSCLC patients. Although considered as an acquired mutation, de novo T790M was reported in many cases [17, 18, 31]. Therefore although rarely detected, de novo T790M could have existed in a very minor part of tumor cells and amplified during EGFR-TKIs treatment. Detection methods for T790M mutation also contributed greatly to the result and might even confound the results. In a study by Fujita et al [31], using a high sensitive assay known as CH (Colony Hybridization), de novo T790M was even found positive in 78.9% of NSCLC TKI-naïve patients. Such results revealed the significance for detection liability. And interestingly, pretreatment T790M was also reported to be related with patients’ outcome. In a meta-analysis of 4 trials, Ding et al. [32] found that advanced NSCLC patients with pre-existed EGFR T790M mutation had a poorer PFS. Thus, the negative prognostic role of pretreatment T790M is different from its favorable implication in NSCLC patients of progression [17, 32].

To our knowledge this is the first meta-analysis to discuss the prognostic role of EGFR T790M in EGFR-TKIs treated NSCLC patients that acquired drug-resistance. Nonetheless, there are several limitations in this study. First of all, the number of the eligible studies was limited. All selected studies were English written; therefore the existing publications in other languages could have been excluded. Due to such constraints, the pooled sample size from individual study was also relatively small. Secondly, several HRs were extracted from survival curves, and the extrapolated HRs might bias the pooled results. Moreover, the clinical stage and the usage of TKIs as first line or multiple line of NSCLC treatment among the eligible studies were incoherent, yet we failed to draw conclusions through subgroup analyses based on above issues due to lack of data. However, with detailed protocol, and carefully pooled statistics, neither publication bias nor heterogeneity was found, the results of the study is guaranteed reliable.

To conclude, T790M is a favorable prognostic factor in EGFR-TKIs treated NSCLC patients that acquired drug resistance. The mechanism of how T790M emerged is complicated, and the prognostic role of T790M in TKIs-naïve patients may differ from secondary T790M mutation in drug resistant patients. The existing publications that focused on the correlation between T790M and NSCLC survival is limited as yet. Future studies are in need to examine the correlation between T790M and clinical outcome of TKIs resistant patients.

## MATERIALS AND METHODS

### Literature search

Two reviewers (GM and JZ) respectively searched on PubMed, Embase, Cochrane Library, China National Knowledge Infrastructure (CNKI) and Wanfang Database up till February 19th, 2017 for relevant literatures. The search items are as followed: “Non-Small Cell Lung Cancer” and “Epidermal Growth Factor Receptor” and “Tyrosine Kinase Inhibitor” and “T790M” and (“Prognosis” or “Outcome” or “Survival”).

### Inclusion criteria

Eligible studies should met all the criteria as followed: 1. Studies on advanced NSCLC patients treated with EGFR-TKIs; 2. T790M mutation was thoroughly examined to discuss the association between acquired mutation existence and survival; 3. Participant should be patients that developed advanced lung cancer; 4. The T790M status should be coherent once detected compared with later mutation assays; 5. Data included among studies should be feasible to calculate the log hazard ratio (logHR) and variance according to methods provided by Parmar, Williamson and Tierney [33–35]. 4. Eligible study types include: cohort study, case-control study and randomized controlled trials (RCT), if any;

### Exclusion criteria

Studies should be excluded if any of the following conditions was matched: 1. Review or systematic review; 2. Case reports; 3. Laboratory studies; 4. Studies without extractable or credible data. 5. T790M assessed through blood (such as ctDNA) without examine NSCLC tumor tissue.

### Data extraction

Basic information extracted was as followed: name of first author, year of publication, country, patient number and gender, number of cases with T790M mutation, smoking history, clinical stage, specimen, histology and treated drugs.

The primary data for calculation was multivariate or univariate Cox hazard regression analysis, the Kaplan-Meier survival curves with P values or hazard ratio (HR) with 95% confidential interval (CI) for overall survival (OS) and progression-free survival (PFS). PFS was defined as the time from the start of TKIs treatment to progressive disease (PD) or death by any cause, and OS was defined as the period from initial use of TKIs, till the death from any cause. The literature selection and data extraction were performed by two reviewers (GM and JZ) independently, with any discrepancies being discussed and reassessed.

### Methodological assessment

Newcastle–Ottawa Scale (NOS) criteria [27] was applied to assess the quality of each study. The NOS scores ranged from 0 to 9, any literature scored 7 or more were considered as a high-quality in the scale. The score evaluated 3 aspects of each study: subject selection: 0 to 4; comparability of subject: 0 to 2; and clinical outcome: 0 to 3. Two reviewers carried out the whole assessment process independently.

### Statistical analysis

The STATA (version 11, Stata Corporation) was used to perform our data analysis. The logHR and variance were extracted for combination of the survival results. If not given directly, the HR with 95% CI or the Kaplan-Meier curves with *P* values were applied for indirect calculation. Adjusted HR was used if adjusted and unadjusted HRs both existed. Multivariate analyses are prior used if univariate and multivariate survival analyses were both provided. Subgroups were divided due to study properties such as regions, clinical stage, smoking history etc. Heterogeneity assumption of pooled HRs was evaluated by chi-square based *Q*-test and I^2^ statistic test [36]. The fixed-effect model (the Mantel-Haenszel method) [37] was used if the heterogeneity between studies was not statistically significant (*P* > 0.10 or I^2^ < 50%). If else, then pooled HR should be evaluated by the random-effect model, to reduce the impact of heterogeneity on the results. The publication bias of pooled studies was assessed according to the methods described by Begg’s et al. [38]. If the *P* value was higher than 0.05 then the publication bias was considered statistically insignificant [39].
